# AN ACTING AND IMPROV CLASS: WELL-BEING AND COMMUNITY BELONGING FOR OLDER AFRICAN AMERICANS IN LOW-INCOME HOUSING

**DOI:** 10.1093/geroni/igz038.520

**Published:** 2019-11-08

**Authors:** Laura Sutherland, Ruth E Dunkle, Garrett T Pace, Ariel Kennedy, Pat Baldwin

**Affiliations:** 1 Wayne State University, Detroit, Michigan, United States; 2 University of Michigan, Ann Arbor, Michigan, United States; 3 Hannan Center, Detroit, Michigan, United States

## Abstract

Arts-based interventions can enhance the quality of life of older adults, but community-dwelling older adults may have reduced access to such interventions. The purpose of this study was to examine whether a creative arts program can improve the overall health and well-being of older adults in low-income housing. A university social work department and community agency collaborated in establishing a professionally run theater group of older adults in two low-income housing buildings in an urban area. All residents were encouraged to participate. The study consisted of three twelve-week acting and improvisation courses, focusing on either staged reading of monologues and dialogues, co-writing a script, or staged reading of a complete play. Each class culminated in a public performance for the building. Fourteen participants enrolled, all African American (11 women, 3 men; mean age 66 years; mean ADL = 1.4 (range of 1-2.5)). Attendance on average was 8 classes. Data were collected through pre- and post-test questionnaires, participant observation of class sessions, semi-structured interviews with building managers, and post-session participant reflections. Thematic analysis was performed, and revealed key themes of increased community belonging, increased confidence, and increased daily coping abilities. Additional themes included the gaining of new artistic skills and interest in continuing classes. Barriers to participation included difficulty in recruitment and absence due to ongoing health conditions and caregiving responsibilities. This project has implications for the potential of arts-based programming to increase well-being for underrepresented community-dwelling older adults.

